# Fatness and fitness: how do they influence health-related quality of life in type 2 diabetes mellitus?

**DOI:** 10.1186/1477-7525-6-110

**Published:** 2008-12-04

**Authors:** Wendy L Bennett, Pamela Ouyang, Albert W Wu, Bethany B Barone, Kerry J Stewart

**Affiliations:** 1 Department of Medicine, Division of General Internal Medicine, Johns Hopkins University School of Medicine, Baltimore, MD, USA; 2 Department of Medicine, Division of Cardiology, Johns Hopkins University School of Medicine, Baltimore, MD, USA; 3 Department of Health Policy and Management, Johns Hopkins Bloomberg School of Public Health, Baltimore, MD, USA; 4 Department of Epidemiology, Johns Hopkins Bloomberg School of Public Health, Baltimore, MD, USA

## Abstract

**Objective:**

We examined whether adiposity and fitness explain the decrease in health-related quality of life (HRQOL) associated with type 2 diabetes mellitus.

**Methods:**

This was a cross-sectional study using baseline data from two exercise training interventions. One study enrolled people with and the other without type 2 diabetes. We assessed aerobic fitness ("fitness") as peak oxygen uptake during treadmill testing, adiposity ("fatness") as percentage of total body fat by dual-energy x-ray absorptiometry, and HRQOL by the Medical Outcomes Study SF-36. Bivariate and multivariate linear regression analyses were used examine determinants of HRQOL were used to examine determinants of HRQOL.

**Results:**

There were 98 participants with and 119 participants without type 2 diabetes. Participants with type 2 diabetes had a mean hemoglobin A1c of 6.6% and, compared with participants without diabetes had lower HRQOL on the physical component summary score (*P *= 0.004), role-physical (*P *= 0.035), vitality (*P *= 0.062) and general health (*P *< 0.001) scales after adjusting for age, sex and race. These associations of HRQOL with type 2 diabetes were attenuated by higher fitness, even more than reduced fatness. Only general health remained positively associated with type 2 diabetes after accounting for fatness or fitness (*P *= 0.003). There were no significant differences between participants with and without diabetes in the mental component score.

**Conclusion:**

Improved fitness, even more than reduced fatness, attenuated the association of type 2 diabetes with HRQOL. The potential to improve HRQOL may motivate patients with type 2 diabetes to engage in physical activity aimed at increasing fitness. Findings from this cross-sectional analysis will be addressed in the ongoing trial of exercise training in this cohort of participants with type 2 diabetes.

**Trial registration:**

NCT00212303

## Background

Type 2 diabetes mellitus (type 2 diabetes) affects approximately 10% of the U.S. population aged 20 years and older and its prevalence increases with age [1]. People with type 2 diabetes report reduced health-related quality of life (HRQOL) compared with the general population, but higher than people with other chronic illnesses such as congestive heart failure [2,3]. The presence of diabetes-related complications, such as peripheral neuropathy, coronary artery disease and peripheral vascular disease, are known to reduce HRQOL [4,5]. Intensive medical treatment regimens may be burdensome to patients and reduce HRQOL [6], but may improve glycemic control and increase net HRQOL [7-9].

Among people with type 2 diabetes, adiposity and reduced fitness have adverse physiological effects that promote disease progression and increase cardiovascular disease mortality [10,11]. Increased adiposity is also an important predictor of HRQOL among people with type 2 diabetes [12,13]. To our knowledge no study has examined the influence of adiposity and fitness on the association of type 2 diabetes and HRQOL, using objective, reproducible measures of adiposity and fitness to compare people with and without diabetes. Based on prior research [12-14], we hypothesized that reduced adiposity and higher fitness levels would attenuate the association of type 2 diabetes with HRQOL. We examined these associations in baseline data from participants recruited for two randomized controlled trials of exercise training for hypertension. For this study we use the term "fatness" to indicate total body adiposity, and "fitness" to indicate cardiorespiratory fitness, which reflects both recent physical activity and genetic makeup [15].

## Methods

The Johns Hopkins Medicine Institutional Review Board (Baltimore, MD) approved both studies. Informed written consent was obtained from each participant. Participants were recruited between 7/1/99 and 11/30/03 for the study in people without type 2 diabetes and between 5/1/04 and 12/28/07 for the study in people with type 2 diabetes.

### Study design and sample

This study was a cross-sectional analysis of baseline data from participants recruited from the local urban and suburban communities for two randomized controlled trials to examine the effect of exercise training on blood pressure. The two studies used similar testing protocols. Each study used newspaper advertising for recruitment. Participants were provided with a cash incentive of $60 for the baseline testing for the study in participants without diabetes and $50 for the study enrolling participants with diabetes.

The first study enrolled people aged 55–75 years old with untreated pre- or stage one hypertension (defined as systolic blood pressure (BP) of 130–159 and/or diastolic BP of 85–99 mm Hg). Subjects who were using only one medicine for hypertension were allowed to discontinue their medicine for 2-weeks prior to undergoing the screening for the study. People with a diagnosis of type 2 diabetes were excluded from the first study.

The second study enrolled people aged 40–65 years old with pre- or stage one hypertension (defined as systolic BP of 130–159 and/or diastolic BP of 85–99 mm Hg) and type 2 diabetes. Because subjects were recruited for an exercise training trial, those with poor glycemic control (fasting blood glucose > 400 mg/dl or HbA1C >11%) or requiring insulin, were excluded. The participants with type 2 diabetes using antihypertensive medications were not discontinued from these medications for the exercise trial, as it was felt that rigorous BP control was indicated in those with a diagnosis of diabetes.

Participants in both studies were sedentary but free of self-reported illnesses, such as chronic pain from orthopedic conditions, peripheral arterial disease and cancer, that could interfere with their full participation in a moderate-intensity exercise program. Both studies excluded people with electrocardiographic abnormalities indicative of myocardial infarction or heart block, smoking and BMI ≥ 40 kg/m^2^. An exercise stress test was used to identify and exclude those with exercise-induced ischemic ST-T wave changes (>1 mm), high-grade arrhythmias, or exercise-induced cardiac symptoms.

### Measures of aerobic fitness, fatness and HRQOL

The protocols for assessing fitness, fatness, and HRQOL were identical in both studies. Fatness was reported as percentage of total body fat measured by dual energy x-ray absorptiometry (DEXA) (GE Lunar Prodigy; General Electric Medical Systems, Milwaukee, Wis).

Aerobic fitness was assessed as the peak oxygen uptake (VO_2_peak), determined on a graded exercise treadmill using a SensorMedics Vmax 229 Metabolic System (SensorMedics Inc, Yorba Linda, Calif). The walking speed was 4.8 km/h at a grade of 0%, and the grade increased by 2.5% every 3 minutes to the point of volitional fatigue, when participants indicated that they could walk no further. Participants were encouraged to reach 18 or higher on the Borg Rating of Perceived Exertion Scale [16].

HRQOL was assessed by the Medical Outcomes Study SF-36 questionnaire [17,18], a self-administered 36-item questionnaire that measures HRQOL using 8 scales: physical function, role limitations due to physical problems (role-physical), role limitations due to emotional problems (role-emotional), vitality, bodily pain, social function, mental health, and general health. Each scale is scored separately from 0 (lowest level of function) to 100 (highest level of function). There are two calculated summary scales, the mental component score and the physical component score. For summary scores, factor weights derived from the U.S. general population were applied to the eight SF-36 scales to compare with a mean of 50 and standard deviation of 10 in the general population [18,19]. The SF-36 has been validated extensively as a measure of health status in people with chronic illness [2] and other settings [20]. The SF-36 has good construct validity, internal consistency and test-retest reliability in racially diverse populations [18,21]. In this study the mean Cronbach's alpha for the eight scales was 0.77 (range 0.71 to 0.83) which is comparable to other studies using the SF-36 [2,20].

### Data Analysis

We pooled baseline data from the two studies to yield a total of 226 participants. Nine participants without a complete SF-36 baseline questionnaire were excluded from analyses. We assessed differences in HRQOL in participants with and without type 2 diabetes using the student's t-test. Because some of the SF-36 scales had distributions that were positively skewed, we confirmed these results using the Mann-Whitney test. We calculated the Spearman correlation coefficient between fatness and fitness. Multivariate linear regression was used to examine the mean difference (represented by the beta coefficient for "presence type 2 diabetes" variable) associated with type 2 diabetes on each HRQOL scale, after adjusting for age, sex and race, potentially confounding variables.

For each HRQOL scale with significant differences associated with type 2 diabetes in either adjusted or unadjusted analyses, additional regression models were created to examine the influence of fatness and fitness on the association of type 2 diabetes with HRQOL. For each HRQOL scale outcome, four models were created. First, we examined the association of type 2 diabetes with each HRQOL scale after controlling for sociodemographics (age, sex and race) in model 1. Model 2 added fatness to model 1 to assess its influence on the association of type 2 diabetes with HRQOL. Model 3 added fitness to Model 1 to test its influence on the type 2 diabetes-HRQOL association. The final model (model 4) added both fatness and fitness to model 1 to examine their combined effect. Interaction terms between fatness and fitness in all final models (model 4) were not statistically significant. Because some of the SF-36 scales did not have normal distributions all regression analyses were repeated using bootstrapping to confirm the reported results. We scaled the partial regression coefficients by about one standard deviation to make them interpretable and comparable across the four models. After scaling, changes in the HRQOL scales in the multivariate models are interpreted per 5 mL/kg·min increase in VO_2_peak and per 10% increase in the percentage of body fat.

Statistical analyses were performed using Stata version 9.2 (College Station, TX) [22].

## Results

### Characteristics of the study participants

In the combined baseline sample there were 217 participants, including 98 participants with and 119 participants without type 2 diabetes. People with type 2 diabetes were younger (p < 0.001), more likely to be male (p = 0.012) and non-white (p < 0.001). Participants with type 2 diabetes had a mean hemoglobin A1c of 6.6% and a lower mean total cholesterol than participants without type 2 diabetes (178.8 mg/dL vs. 215.2 mg/dL) (Table 1).

**Table 1:** Characteristics of and health-related quality of life in participants with and without type 2 diabetes

**Characteristic**	**No type 2 Diabetes** **N = 119**	**With type 2 Diabetes** **N = 98**	***P *value**
Mean age (SD), years	63.8 (5.6)	56.9 (5.9)	< 0.001
Men, %	46.2	63.3	0.012
White race, %	85.7	60.2	< 0.001
Mean percentage of body fat (SD), %	38.2 (9.2)	36.0 (7.1)	0.068
Mean VO_2_peak (SD), mL/kg·min	24.1 (5.1)	21.2 (5.1)	< 0.001
Mean total cholesterol level (SD), mg/dL	215.2 (37.5)	178.8 (40.2)	< 0.001
Mean hemoglobin A1c (SD), %	N/A	6.6 (1.5)	N/A
			
**SF-36 HRQOL Scale**	**Mean (SD)**	**Mean (SD)**	***P *value**
Bodily pain	78.0 (17.9)	74 (16.4)	0.130
General health	80.3 (14.1)	65.7 (17.0)	< 0.001
Mental health	84.3 (11.7)	82.0 (14.2)	0.189
Physical function	86.4 (13.5)	85.6 (14.2)	0.656
Role emotional	87.7 (24.5)	84.7 (28.8)	0.411
Role physical	89.3 (20.0)	82.8 (29.1)	0.055
Social function	93.3 (12.9)	89.3 (18.8)	0.066
Vitality	68.8 (16.1)	63.5 (19.5)	0.028
Physical component score	51.1 (5.6)	48.4 (6.9)	0.002
Mental component score	54.8 (6.9)	53.3 (8.8)	0.156

Abbreviations: SD = standard deviation; HRQOL = health-related quality of life; kg = kilogram; m = meter; mL = millileter; min = minute.

Participants without type 2 diabetes had a higher mean percentage of body fat (38.2% vs. 36.0%) (Table 1). This difference was explained by a higher percentage of females without type 2 diabetes (53.7% vs. 37.3%), as women had a higher percentage of body fat versus men (mean of 44% vs. 32%, respectively). When stratified by sex there was no significant difference in percent body fat between participants with and without type 2 diabetes. Participants with type 2 diabetes also had lower levels of VO_2_peak despite being younger (Table 1).

Fatness and fitness were negatively correlated, with the Spearman *r *= -0.6241 (p < 0.001).

### Comparison of HRQOL scales in participants with and without type 2 diabetes

Participants with type 2 diabetes had lower mean scores for general health (p < 0.001), vitality (p = 0.028) and the physical component summary (p = 0.002) scales. The mean SF-36 scales for bodily pain, physical function, role emotional, role physical, social function, mental health and mental component score were all lower in people with type 2 diabetes but the differences did not reach statistical significance (Table 1).

For each HRQOL scale, we assessed the mean difference in HRQOL associated with the presence of type 2 diabetes, after adjusting for age, sex and race (Table 2). Type 2 diabetes was associated with a 12-point decrease in general health (*P*< 0.001), 8-point decrease in role limitations due to physical problems (*P *= 0.035), 5-point decrease in vitality and almost a 3-point decrease in the physical component score (Table 2).

**Table 2:** Adjusted* mean differences in SF-36 HRQOL scales associated with type 2 diabetes

**SF-36 HRQOL Scale**	Adjusted* mean differences associated with type 2 diabetes	***P *value**
Bodily pain	-5.28	0.065
General health	-12.84	< 0.001
Mental health	-1.84	0.388
Physical function	-1.28	0.572
Role emotional	-4.86	0.259
Role physical	-8.61	0.035
Social function	-2.83	0.282
Vitality	-5.48	0.062
Physical component score	-2.99	0.004
Mental component score	-1.33	0.300

Abbreviations: HRQOL = health-related quality of life;

* Adjusted for age, sex and race.

### Influence of fatness and fitness on the differences in HRQOL associated with type 2 diabetes

We assessed the influence of fatness and fitness individually and then together on the differences in HRQOL associated with type 2 diabetes in the general health, role physical and vitality scales, and the physical component score. These scales had significant differences associated with type 2 diabetes in either unadjusted analyses or analyses adjusted for age, sex and race.

For general health, the addition of fatness to model 1 did not greatly influence the decrement in HRQOL associated with type 2 diabetes (mean difference of -12.87, *P *= 0.001). Fitness influenced the association of general health with type 2 diabetes (models 3 and 4), but type 2 diabetes remained highly significant [Additional file 1]. An improvement in fitness by 5 mL/kg·min of VO_2_peak was associated with a 4-point increase in general health in the final model [Additional file 1].

Type 2 diabetes was associated with an 8-point decrease in the role-physical scale after adjusting for sociodemographic characteristics and fatness (*P *= 0.035, model 2). The negative association of type 2 diabetes with the role-physical scale was attenuated, becoming non-significant, with the addition of fitness in models 3 and 4 [Additional file 1].

The addition of fitness in models 3 and 4 attenuated the association of type 2 diabetes and vitality. An improvement in fitness by 5 mL/kg·min of VO_2_peak was associated with a 4-point increase in vitality in model 4 [Additional file 1].

The association of type 2 diabetes with the physical component score was augmented with the addition of fatness in mode1 2 (mean difference of -3.25, *P *= 0.002). A 10% increase in the percentage of body fat was associated with a 2-point decrease in the physical component score (model 2). However, the negative association between type 2 diabetes and the physical component score was attenuated, becoming non-significant, with the addition of fitness in models 3 and 4 [Additional file 1].

## Discussion

There are several important new findings in this cross-sectional study examining the influence of fatness and fitness on the association of type 2 diabetes with HRQOL in participants with and without type 2 diabetes. First, we confirmed the negative impact on the physical aspects of HRQOL in type 2 diabetes, which was concentrated in the scales measuring role limitations due to physical problems (role-physical), vitality, general health, and the physical component summary score. No significant reductions were found in self-reported mental health in our participants, who on-average, had well-controlled type 2 diabetes. Second, a new finding from this study was that higher levels of fitness, more so than lower fatness, attenuated much of the association of HRQOL with type 2 diabetes in most of these physical health domains. However, neither fatness nor fitness ameliorated the strong negative association of type 2 diabetes with the general health scale. This seems reasonable, since the presence of a diabetes diagnosis in and of itself would be expected to influence a patient's self perceived health.

Few studies have examined the association of fatness and fitness on HRQOL in people with type 2 diabetes. In the Look Ahead Study, both lower fitness and obesity were associated with lower physical component summary scores in people with type 2 diabetes [13], a finding consistent with our study. In our study fatness and fitness are associated with each of the eight SF-36 scales. We provide insight about how fatness and fitness may affect general, mental and physical health individually. This was especially informative in identifying a significant association of type 2 diabetes with general health, even after adjusting for fatness and fitness. It is notable that we found no significant association between type 2 diabetes and reduced mental health, despite studies showing an increase in the prevalence of depression among people with type 2 diabetes [23]. This may reflect that the present participants volunteered for an exercise program, suggesting an interest in improving their health, and had well-controlled diabetes, thus less likely to be experiencing symptoms and disease complications that might impact their mental health.

There are limitations to our study. First, because the participants were volunteers for exercise training studies and had well-controlled type 2 diabetes, they were, by definition, less likely to have diabetes-related complications and co-morbid illnesses. Nevertheless, unmeasured less severe co-morbidities such as arthritis and use of medications may be different between the two studies, which could be confounders. Importantly, prior studies have found that disease complications are the strongest determinants of quality of life in people with type 2 diabetes [3]. However, the studies' exclusion criteria make it more likely that the two groups were otherwise similar with respect to health status, except for their diabetes diagnosis. A second limitation is that the two combined studies used slightly different selection criteria for age (55–75 years in the study without diabetes and 40–65 years in the study with diabetes) making age a potential confounder. To address this limitation, we performed a sensitivity analysis in the sample with overlapping ages (N = 143) and confirmed the overall results. A third limitation is that the two combined study populations differed with respect to the use of antihypertensive therapy, which may also be a confounder. Both populations had the diagnosis of pre- or stage 1 hypertension, but those without diabetes were untreated according to the study protocol; whereas, those with diabetes were continued on the antihypertensive medications prescribed by their own health care providers. This is unlikely to influence our results, as both groups do meet criteria for pre- or stage 1 hypertension, and compared with other chronic diseases, hypertension has been shown to have the lowest impact on quality of life [2]. A fourth limitation was the use of the SF-36, a general not diabetes-specific, HRQOL instrument. It may have been less responsive to diabetes-specific symptoms and aspects of life [24,25]. The SF-36 was used as it is both reliable and valid in these populations, and allowed for comparisons between groups with and without type 2 diabetes. However, many studies of HRQOL in people with type 2 diabetes are now including both a diabetes-specific and a general measure of HRQOL [12,26]. Finally, the sample size of 217 may have reduced our power to detect differences in HRQOL, especially in the area of mental health, where the differences were not statistically significant.

There are several notable strengths to our study. The selection criteria enabled us to recruit people with less-complicated type 2 diabetes, who may represent a large number of patients, such as those with new diagnoses and not requiring insulin. These patients may also be more physically able, as well as motivated to engage in lifestyle changes. Despite having well-controlled disease, we did detect significant HRQOL detriments as compared to the participants without type 2 diabetes. Another study strength was the use of two reproducible and precise measures of fatness and fitness, namely the percentage of body fat obtained from DEXA and the VO_2_peak obtained from treadmill testing. Other studies, including the Look Ahead study used estimated MET capacity, as the fitness measure, and body mass index, a crude measure of total adiposity [13].

This study has several public health and clinical implications. It is well-established that successful adoption of health behaviors focused on diet and exercise leading to weight loss and increased fitness improve clinical outcomes in people with type 2 diabetes [27,28]. These results suggest that higher levels of fitness might also enhance HRQOL. This is especially important because studies show that lower HRQOL is independently associated with both cardiovascular events [29] and higher mortality in people with type 2 diabetes [30]. Providers may be motivated to more frequently assess HRQOL. In addition, providers might counsel patients with type 2 diabetes that incorporating physical activity into their daily routine improves fitness, allows them to do more, feel better, as well as reduce their risk of cardiovascular disease and diabetes-related complications.

Further studies are needed to confirm our findings from this cross-sectional study. There have been few studies examining the effect of a lifestyle intervention, such as exercise, on HRQOL in people with type 2 diabetes [31]. We anticipate having results in late 2009 for the exercise training trial in participants with type 2 diabetes to be able assess whether exercise leading to improved fitness levels improves HRQOL, as our cross-sectional results suggest. Future studies could examine the effects of fatness and fitness in a population with a wider range of diabetes severity, treatment types and co-morbid illness, in order to control for the multitude of factors that impact HRQOL. In addition, it would be helpful to explore the presence of depressive symptoms and use both general and diabetes-specific instruments to better understand the relationships between fitness, fatness and HRQOL in type 2 diabetes.

## Conclusion

Uncomplicated type 2 diabetes is associated with lower HRQOL. Improved fitness, even more than reduced fatness, was associated with improved HRQOL in people with type 2 diabetes. Ongoing research is addressing whether or not increased fitness levels improve HRQOL. Further investigation is needed to assess the role of fitness and fatness on HRQOL in populations with a wider range of diabetes-related complications and co-morbid illnesses.

## Abbreviations

BP: blood pressure; DEXA: dual-energy x-ray absorptiometry; Fatness: adiposity; Fitness: aerobic fitness; HgBA1c: hemoglobin A1c; HRQOL: health-related quality of life; kg: kilogram; m: meter; mL: millileter; min: minute; SD: standard deviation; Type 2 diabetes: type 2 diabetes mellitus; T2DM: type 2 diabetes mellitus; VO_2_peak: peak oxygen uptake determined at volitional fatigue during treadmill testing.

## Competing interests

The authors declare that they have no competing interests.

## Authors' contributions

WB contributed to the development of the research question, study planning, data analysis and drafting and revising the manuscript. PO contributed to the study design, development of the research question and revising the manuscript and has given final approval for the version to be published. AW contributed to the development of the analytic plan and editing the manuscript has given final approval for the version to be published. BB contributed to the development of the analytic plan and editing the manuscript and has given final approval for the version to be published. KS was the principal investigator for the two trials that this study is based on, and as such, contributed to study design, data collection, development of the research question, study planning and manuscript editing. He has given final approval for the version to be published.

## Supplementary Material

Additional file 1 Table 3. Influence of fatness and fitness on the association of type 2 diabetes with health-related quality of life. The data represent the multivariate regression models for four HRQOL outcomes to examine the influence of fatness and fitness on the association of type 2 diabetes with HRQOL. Click here for file

## References

[B1] National Institute of Diabetes and Digestive and Kidney Diseases (2005). National Diabetes Statistics fact sheet: General information and national estimates on diabetes in the United States.

[B2] Stewart AL, Greenfield S, Hays RD, Wells K, Rogers WH, Berry SD, McGlynn EA, Ware JE (1989). Functional status and well-being of patients with chronic conditions. Results from the Medical Outcomes Study. JAMA.

[B3] Rubin RR, Peyrot M (1999). Quality of life and diabetes. Diabetes Metab Res Rev.

[B4] Glasgow RE, Ruggiero L, Eakin EG, Dryfoos J, Chobanian L (1997). Quality of life and associated characteristics in a large national sample of adults with diabetes. Diabetes Care.

[B5] Wandell PE (2005). Quality of life of patients with diabetes mellitus. An overview of research in primary health care in the Nordic countries. Scand J Prim Health Care.

[B6] Jacobson AM, de Groot M, Samson JA (1994). The evaluation of two measures of quality of life in patients with type I and type II diabetes. Diabetes Care.

[B7] Menard J, Payette H, Dubuc N, Baillargeon JP, Maheux P, Ardilouze JL (2007). Quality of life in type 2 diabetes patients under intensive multitherapy. Diabetes Metab.

[B8] (1999). Quality of life in type 2 diabetic patients is affected by complications but not by intensive policies to improve blood glucose or blood pressure control (UKPDS 37). U.K. Prospective Diabetes Study Group. Diabetes Care.

[B9] Jacobson AM (2004). Impact of improved glycemic control on quality of life in patients with diabetes. Endocr Pract.

[B10] Wei M, Gibbons LW, Kampert JB, Nichaman MZ, Blair SN (2000). Low cardiorespiratory fitness and physical inactivity as predictors of mortality in men with type 2 diabetes. Ann Intern Med.

[B11] Church TS, LaMonte MJ, Barlow CE, Blair SN (2005). Cardiorespiratory fitness and body mass index as predictors of cardiovascular disease mortality among men with diabetes. Arch Intern Med.

[B12] Sundaram M, Kavookjian J, Patrick JH, Miller LA, Madhavan SS, Scott VG (2007). Quality of life, health status and clinical outcomes in Type 2 diabetes patients. Qual Life Res.

[B13] Rejeski WJ, Lang W, Neiberg RH, Van Dorsten B, Foster GD, Maciejewski ML, Rubin R, Williamson DF, Look AHEAD Research Group (2006). Correlates of health-related quality of life in overweight and obese adults with type 2 diabetes. Obesity (Silver Spring).

[B14] Stewart KJ, Turner KL, Bacher AC, DeRegis JR, Sung J, Tayback M, Ouyang P (2003). Are fitness, activity, and fatness associated with health-related quality of life and mood in older persons?. J Cardiopulm Rehabil.

[B15] Haskell WL, Leon AS, Caspersen CJ, Froelicher VF, Hagberg JM, Harlan W, Holloszy JO, Regensteiner JG, Thompson PD, Washburn RA (1992). Cardiovascular benefits and assessment of physical activity and physical fitness in adults. Med Sci Sports Exerc.

[B16] Borg G (1998). Measuring Perceived Exertion and Pain. Borg's Perceived Exertion and Pain Scales.

[B17] Stewart AL, Ware JE (1992). Measuring Functioning and Well-being: The Medical Outcomes Study Approach.

[B18] Ware JE, Snow KK, Kosinski M (1993). SF-36 Health Survey: Manual and Interpretation.

[B19] Ware JE, Kosinski M (2001). SF-36 physical & mental health summary scales: A manual for users of version 1.

[B20] Brazier JE, Harper R, Jones NM, O'Cathain A, Thomas KJ, Usherwood T, Westlake L (1992). Validating the SF-36 health survey questionnaire: new outcome measure for primary care. BMJ.

[B21] McHorney CA, Ware JE, Lu JF, Sherbourne CD (1994). The MOS 36-item Short-Form Health Survey (SF-36): III. Tests of data quality, scaling assumptions, and reliability across diverse patient groups. Med Care.

[B22] StataCorp (2005). Stata Statistical Software Release 9.

[B23] Anderson RJ, Freedland KE, Clouse RE, Lustman PJ (2001). The prevalence of comorbid depression in adults with diabetes: a meta-analysis. Diabetes Care.

[B24] Polonsky WH, Fisher L, Earles J, Dudl RJ, Lees J, Mullan J, Jackson RA (2005). Assessing psychosocial distress in diabetes: development of the diabetes distress scale. Diabetes Care.

[B25] El Achhab Y, Nejjari C, Chikri M, Lyoussi B (2008). Disease-specific health-related quality of life instruments among adults diabetic: A systematic review. Diabetes Res Clin Pract.

[B26] Huang IC, Hwang CC, Wu MY, Lin W, Leite W, Wu AW (2008). Diabetes-specific or generic measures for health-related quality of life? evidence from psychometric validation of the D-39 and SF-36. Value Health.

[B27] Sigal RJ, Kenny GP, Boule NG, Wells GA, Prud'homme D, Fortier M (2007). Effects of aerobic training, resistance training, or both on glycemic control in type 2 diabetes: a randomized trial. Ann Intern Med.

[B28] Hamdy O, Goodyear LJ, Horton ES (2001). Diet and exercise in type 2 diabetes mellitus. Endocrinol Metab Clin North Am.

[B29] Hayes AJ, Clarke PM, Glasziou PG, Simes RJ, Drury PL, Keech AC (2008). Can self-rated health scores be used for risk prediction in patients with type 2 diabetes?. Diabetes Care.

[B30] Kleefstra N, Landman GW, Houweling ST, Ubink-Veltmaat LJ, Logtenberg SJJ, Meyboom-DeJong B, Coyne JC, Groenier KH, Bilo HJG (2008). Prediction of mortality in type 2 diabetes from health-related quality of life (ZODIAC-4). Diabetes Care.

[B31] Zhang X, Norris SL, Chowdhury FM, Gregg EW, Zhang P (2007). The effects of interventions on health-related quality of life among persons with diabetes: a systematic review. Med Care.

