# Supplementary material for: Fatness and fitness: how do they influence health-related quality of life in type 2 diabetes mellitus?
Source: Health Qual Life Outcomes. 2008 Dec 4;6:110. doi: 10.1186/1477-7525-6-110 (PMC2626587; doi:10.1186/1477-7525-6-110)
Supplement: Additional file 1 — Table 3. Influence of fatness and fitness on the association of type 2 diabetes with health-related quality of life. The data represent the multivariate regression models for four HRQOL outcomes to examine the influence of fatness and fitness on the association of type 2 diabetes with HRQOL. [file 1477-7525-6-110-S1.doc]

**Additional File 1**

**Table 3. Influence of fatness and fitness on the association of type 2 diabetes with health-related quality of life***

| **HRQOL scale** | **Independent**  **Variables** | Model 1 | Model 2 | Model 3 | Model 4 |
| --- | --- | --- | --- | --- | --- |
|  |  | **T2DM + sociodemographics**** | **T2DM + sociodemographics** + fatness** | **T2DM + sociodemographics** + fitness** | **T2DM + sociodemographics** + fatness + fitness** |
| General health  mean difference  (*P-*value) | T2DM Diagnosis | **- 12.84 (<0.001)** | **- 12.87 (0.001)** | **- 9.60 (0.001)** | **- 9.18 (0.003)** |
|  | Fatness  (per 10% increase in % body fat) |  | - 2.06 (0.262) |  | - 0.80 (0.703) |
|  | Fitness  (per 5 mL/kg·min increase in VO_2_peak |  |  | **+ 4.14 (0.003)** | **+ 4.33 (0.009)** |
| Role physical  mean difference  (*P-*value) | T2DM Diagnosis | **- 8.61 (0.035)** | **- 8.80 (0.035)** | - 4.12 (0.369) | - 4.45 (0.359) |
|  | Fatness  (per 10% increase in % body fat) |  | - 2.93 (0.306) |  | - 0.34 (0.919) |
|  | Fitness  (per 5 mL/kg·min increase in VO_2_peak |  |  | **+ 4.88 (0.030)** | + 4.42 (0.090) |
| Vitality  mean difference  (*P-*value) | T2DM Diagnosis | - 5.48 (0.062) | - 4.73 (0.106) | - 1.77 (0.587) | - 1.00 (0.769) |
|  | Fatness  (per 10% increase in % body fat) |  | - 3.12 (0.123) |  | - 0.51 (0.825) |
|  | Fitness  (per 5 mL/kg·min increase in VO_2_peak |  |  | **+ 4.43 (0.006)** | **+ 4.11 (0.024)** |
| Physical component score  mean difference  (*P-*value) | T2DM Diagnosis | **- 2.99 (0.004)** | **- 3.25 (0.002)** | - 1.29 (0.253) | - 2.13 (0.088) |
|  | Fatness  (per 10% increase in % body fat) |  | **- 2.30 (0.001)** |  | - 1.53 (0.059) |
|  | Fitness  (per 5 mL/kg·min increase in VO_2_peak |  |  | **+ 1.92 (0.001)** | **+ 1.25 (0.049)** |

* HRQOL scales were limited to those showing significant differences between participants with and without T2DM and in prior unadjusted or adjusted analyses.

** sociodemographics adjusted for were age, sex and race

Abbreviations: HRQOL= health-related quality of life; T2DM= Type 2 diabetes mellitus
